# Mesenchymal Stem Cells Reshape and Provoke Proliferation of Articular Chondrocytes by Paracrine Secretion

**DOI:** 10.1038/srep32705

**Published:** 2016-09-06

**Authors:** Lei Xu, Yuxi Wu, Zhimiao Xiong, Yan Zhou, Zhaoyang Ye, Wen-Song Tan

**Affiliations:** 1 State Key Laboratory of Bioreactor Engineering, East China University of Science and Technology, Shanghai 200237, China

## Abstract

Coculture between mesenchymal stem cells (MSCs) and articular chondrocytes (ACs) represents a promising strategy for cartilage regeneration. This study aimed at elaborating how ACs were regulated by MSCs. Rabbit ACs (rACs) and rabbit MSCs (rMSCs) were seeded separately in a Transwell system to initiate non-contact coculture in growth medium without chondrogenic factors. Cell morphology, cell proliferation, production of extracellular matrix (ECM), and gene expression of rACs were characterized. Upon coculture, rACs underwent a morphological transition from a rounded or polygonal shape into a fibroblast-like one and proliferation was provoked simultaneously. Such effects were dependent on the amount of rMSCs. Along with these changes, ECM production and gene expression of rACs were also perturbed. Importantly, when a ROCK inhibitor (Y27632) was supplemented to coculture, the effects except that on cell proliferation were inhibited, suggesting the involvement of RhoA/ROCK signaling. By applying an inhibitor (BIBF1120) of VEGFR1/2/3, FGFR1/2/3 and PDGFRα/β in coculture, or supplementing FGF-1, VEGF-A and PDGFbb in monoculture, it was confirmed that the paracrine factors by rMSCs mediated the compounding effects on rACs. These findings shed light on MSCs-ACs interactions and might confer an insight view on cell-based cartilage regeneration.

Articular cartilage has limited self-repair capability. Current clinical treatments such as microfracture, a surgical technology drilling the subchondral bone, making bone marrow enter the damaged area to promote tissue repair, have generally led to a mechanically inferior fibrocartilage, which eventually undergoes recurrent degeneration. Subsequently, novel cell-based strategies have been developed and are expected to solicit cartilage tissue regeneration^1^. One of these, so-called “autologous chondrocyte implantation” (ACI), applies *ex vivo* expanded autologous chondrocytes in collagen matrix to the lesion site and has been translated to clinic. Alternatively, tissue engineering by seeding cells into biomaterial scaffolds to fabricate off-the-shelf tissue replacements represents another route for cartilage repair. In both methodologies, chondrocytes and mesenchymal stem cells (MSCs) have been extensively exploited, which are however associated with several critical issues^1^. While chondrocytes inevitably undergo the unfavorable dedifferentiation (loss of phenotype) during *ex vivo* expansion, MSCs in current chondrogenic induction protocols tend to express a hypertrophic phenotype and subsequent calcification^2^,^3^.

Recently, coculturing MSCs and chondrocytes has emerged as a promising strategy that permitting cell-cell interactions between the two cell types to solicit better cartilage repair^4^,^5^,^6^,^7^,^8^. The rationale is that in a coculture system the advantages of these two cell types can be exploited. Articular chondrocytes (ACs) bear a desired cartilage phenotype and can secrete abundant cartilaginous extracellular matrix (ECM), and MSCs possessing the differentiation potential are readily available in a great amount via *in vitro* expansion^9^. Consequently, through such a coculture strategy, on one hand, the demand for a large quantity of primary chondrocytes can be attenuated by substituting with MSCs; on the other hand, mature ACs are anticipated to confer instructive cues for the chondrogenesis of neighbouring MSCs to obtain a hyaline cartilage phenotype. This feasibility has been tested in several studies. Hildner *et al*. investigated the effects of partially substituting human ACs with human adipose-derived MSCs on chondrogenesis in matrix-associated ACI^10^; Chen *et al*. showed that human MSCs could undergo a stage-specific chondrogenic differentiation in non-contact coculture with an immortalized human ACs cell line^11^.

However, subsequent intensive research efforts have generated conflicting findings concerning coculture of MSCs and chondrocytes, indicating that interactions between these two cell types are far more complicated than initially thought^6^,^7^. At first, many studies have demonstrated that the chondrogenic differentiation of MSCs can be stimulated by coculturing with ACs^11^,^12^,^13^,^14^ and a hypertrophic phenotype can be potentially inhibited, conferring the hope to generate a hyaline cartilage^15^,^16^,^17^. Later on, chondrogenesis of MSCs is found to be absent in some coculture studies and a novel view that MSCs can exert trophic effects on cocultured chondrocytes is developed, instead^18^,^19^,^20^,^21^,^22^. Accordingly, the apparent improvement of chondrogenesis in certain coculture systems is claimed to be derived from both improved differentiation of MSCs and stimulated chondrocyte proliferation and/or ECM production, emphasizing the bidirectional interactions between cocultured two cell types^23^,^24^. Moreover, very recently, Lee *et al*. reported that costochondral chondrocytes cocultured with MSCs displayed a significant reduction in chondrocytic gene expression and proteoglycan production^25^. Consistently, we previously had also observed that MSCs could significantly downregulate the phenotype of ACs in a Transwell-based non-contact coculture system^26^. Thus, a further comprehensive elaboration on MSCs-ACs interactions would be necessary for developing regenerative therapies for cartilage based on coculturing MSCs and ACs.

The objective of the present work was to gain an insight understanding of how ACs were regulated by MSCs in coculture. As illustrated in Fig. 1, a non-contact coculture model via a Transwell insert was applied and MSCs and ACs were plated in the insert and at the bottom of tissue culture plate well, respectively. Both phenotype and gene expression were examined for ACs after coculture with MSCs. Potential signaling pathways were interrogated to explore the molecular mechanisms mediating MSCs-ACs interactions.

## Results

### rMSCs reshaped rACs in coculture

F-actin staining was performed to characterize cell morphology. As shown in Fig. 2A, coculture induced a drastic morphological change of rACs within 48 h compared to those in control monoculture and cells tended to display a spindle-like shape in coculture. At 8 h post coculture, the spindle-like cells could be discerned in coculture. As coculture proceeded, more cells underwent the transformation. Such a phenomenon also appeared to be dependent on the density of cocultured rMSCs and at an increasing density of rMSCs, the shape change became more prominent. F-actin staining images at 48 h post coculture were further analyzed to determine cell elongation factor and roundness. As the presence of rMSCs, cell elongation factor of rACs increased compared to that of monocultured cells (Fig. 2B) and cell roundness decreased (Fig. 2C). The density-dependent trend was also confirmed. Such a morphological transition of rACs in coculture was further confirmed with scanning electron microscopy (Figure S1) as well as immunofluorescence staining of other cytoskeletal proteins including α-tubulin and vimentin (Figure S2). More importantly, when rACs were cultured in rMSCs-conditioned medium, a similar change in shape could be achieved (Figure S3).

### Proliferation of rACs was provoked by rMSCs in coculture

An EdU assay was performed to evaluate the proliferation of rACs in coculture. Positive EdU staining displayed purple and cell nuclei were counterstained with DAPI (blue). As shown in Fig. 3A, at 24 h post coculture, the number of EdU^+^ cells dramatically increased in coculture compared to that in control monoculture. As the density of cocultured rMSCs increased, more positively stained rACs were observed. Based on the imaging analysis, a quantitative comparison showed that the frequency of EdU^+^ cells was significantly higher when cocultured with rMSCs at 5 × 10^5^ and 1 × 10^6^ cells/well compared to that in monoculture (Fig. 3B).

After 6 days of coculture, rACs were harvested and counted. As shown in Fig. 3C, the number of rACs in coculture with rMSCs at a density of ≥2.5 × 10^5^ cells/well was significantly higher than that in monoculture and increased as the density of cocultured rMSCs increased. It was of note that the number of rACs in coculture with rMSCs at 1 × 10^6^ cells/well was almost twice as that in monoculture.

### Y27632 restrained the morphological change, rescued ECM production and reverted gene expression of rACs in coculture

To investigate whether Ras homolog gene family, member A (RhoA)/Rho-associated, coiled-coil containing protein kinase (ROCK) pathway was involved in such a morphological change^27^, ROCK inhibitor Y27632 was added in coculture. F-actin staining of rACs was performed on day 4 post coculture. At the presence of Y27632 in coculture, F-actin retained a radial distribution in rACs at all tested densities of rMSCs, resembling that in monoculture, but distinct from the aligned arrangement in coculture at the absence of Y27632 (Fig. 4A). Staining of α-tubulin and vimentin also showed that rACs in coculture at the presence of Y27632 remained the morphological characteristics as in monoculture (Figure S2). And, supplementation of Y27632 inhibited the shape change of rACs upon culture in rMSCs-conditioned medium (Figure S3). These results suggested that Y27632 could prevent the morphological transformation of rACs induced by coculture with rMSCs.

Accompanying the morphological characterization, deposition of cartilaginous ECM including type II collagen and glycosaminoglycans (GAG) by cocultured rACs was also assessed. In coculture, production of both type II collagen (immunofluorescence staining) and GAG (safranin O staining) by rACs was dramatically reduced on day 6 post coculture and a higher density of rMSCs in coculture led to a more intensive reduction (Fig. 4A). In contrast, when Y27632 was supplemented in coculture, deposition of both type II collagen and GAG could be rescued, especially in coculture at low densities of rMSCs. Quantification of GAG content further confirmed this observation and, GAG/DNA in coculture with rMSCs at 2 × 10^4^ cells/well could even be recovered by supplementing Y27632 to a similar level as that in monoculture (Fig. 4B). Meanwhile, DNA content was not significantly different between coculture with and without Y27632 (Fig. 4B), which was also confirmed by cell counting (Figure S4).

Gene expression of rACs was further analyzed. Compared to those in monoculture, genes including *Sox9, Acan, Col2a1, Fn1, CD44* and *Thy1* were downregulated in coculture, while *Vcan, Col1a1, CD14, ROCK1* and *ROCK2* were upregulated (Fig. 4C). Most integrin genes including *Itga1, Itga2, Itga5, Itgav, Itgb1*, and *Itgb5* were upregulated in coculture, and only *Itga10* was downregulated. Additionally, *Itga6* remained constant and *Itgb3* was not detected at all in rACs in all conditions. When Y27632 was added in coculture, such changes in gene expression induced by coculture were inhibited and gene expression maintained at levels close to those in monoculture.

### BIBF1120 abolished the effects of rMSCs on rACs in coculture

BIBF1120, an inhibitor of vascular endothelial growth factor receptor-1/2/3 (VEGFR1/2/3), fibroblast growth factor receptor-1/2/3 (FGFR1/2/3) and platelet-derived growth factor receptor-α/β (PDGFRα/β) receptors, was added in coculture to test whether the modulatory effects of rMSCs on rACs could be attributed to vascular endothelial growth factor (VEGF), fibroblast growth factor (FGF) and platelet-derived growth factor (PDGF). BIBF1120 turned rACs in coculture into a polygonal shape, akin to that in monoculture, rather than the spindle-like morphology displayed in coculture without BIBF1120 (Fig. 5A). In rMSCs-conditioned medium, BIBF1120 also maintained rACs in a round morphology (Figure S5). It was also found that the number of rACs was much less in BIBF1120-treated coculture compared to those in both monoculture and coculture without BIBF1120 (Fig. 5B). GAG/DNA was significantly promoted at the presence of BIBF1120 in coculture, reaching a level even higher than that in monoculture (Fig. 5B). Upon addition of BIBF1120 in coculture, expression of genes including *Col1a1, Col2a1, CD14, Thy1 Itga1, Itga2, Itga5, Itga6, Itga10, Itgav, Itgb1* and *Itgb5* was maintained at levels more close to that in monoculture (Fig. 5C). Further, when a specific inhibitor of FGFR1, PD173074, was supplemented in coculture (Figure S6) or culture in rMSCs-conditioned medium (Figure S5), the changes in both shape and proliferation of rACs were inhibited, albeit being less efficient than BIBF1120.

### A cocktail of VEGF-A, FGF-1 and PDGFbb recapitulated the effects of rMSCs on rACs

To further confirm the involvement of the paracrine factors secreted by rMSCs, growth factors including VEGF-A, FGF-1 and PDGFbb either in individual or in combination were supplemented in monoculture of rACs. As shown in Fig. 6A, while VEGF-A did not induce any morphological change of rACs, FGF-1 and PDGFbb resulted in slightly variation in the organization of F-actin. Strikingly, combinations of FGF-1&VEGF-A and FGF-1&VEGF-A&PDGFbb turned rACs into the spindle-like shape, mimicking that in coculture, especially at high densities of rMSCs. Additionally, cell number in culture with FGF-1, PDGFbb and FGF-1&VEGF-A&PDGFbb was significantly higher than that in control without growth factor supplementation (Fig. 6B).

Compared to control, total GAG content produced by rACs was significantly inhibited with all different conditions and GAG/DNA was found significantly lower in culture with FGF-1, FGF-1&VEGF-A and FGF-1&VEGF-A&PDGFbb (Fig. 6B). Gene expression of rACs in culture with growth factor supplementation was also analyzed. In general, different growth factors induced varied changes in gene expression compared to control (Fig. 6C). Specifically, with all kinds of growth factor supplementation, expression of *Sox9* and *CD14* was slightly promoted compared to that in control culture. While FGF-1 and FGF-1&VEGF-A&PDGFbb tended to downregulate the expression of *Col1a1, Col2a1, COMP, CD44* and *Thy1*, these genes except *CD44* were slightly upregulated at the presence of VEGF-A and FGF-1&VEGF-A. In addition, FGF-1 and FGF-1&VEGF-A increased the expression of *Acan*, and VEGF-A, PDGFbb and FGF-1&VEGF-A apparently stimulated the expression of *Vcan*. For integrins, FGF-1 and FGF-1&VEGF-A&PDGFbb slightly inhibited the expression of *Itga5, Itga10, Itgav, Itgb1* and *Itgb5*, which were upregulated by VEGF-A, PDGFbb and FGF-1&VEGF-A. Expression of *Itga1, Itga2* and *Itga6* was slightly stimulated with growth factor supplementation. When these growth factors individually or in combination were supplemented at a doubled dose, similar trends but with more intensified effects in cell morphology, proliferation, GAG production and gene expression were observed (Figure S7).

To further validate the effects of these growth factors on rACs, these factors were also supplemented in serum-free medium and applied in monoculture of rACs for 6 days. As shown in Figure S8, elongated cells could be observed at the presence of PDGFbb and FGF-1&VEGF-A&PDGFbb. Cell number was significantly higher and GAG/DNA was significantly lower at the presence of FGF-1, PDGFbb, FGF-1&VEGF-A and FGF-1&VEGF-A&PDGFbb when compared to control without growth factor supplementation. These results essentially confirmed the potential involvement of these factors in the paracrine effects of rMSCs.

## Discussion

In the present study, rMSCs were demonstrated to induce a drastic morphological change and at the same time stimulate proliferation of rACs in the non-contact coculture. These changes were associated with reduction in chondrocytic gene expression (*Sox9, Col2a1*, and *Acan*) and ECM production (type II collagen and GAG). However, gene expression of integrins and other surface molecules was also perturbed by coculture. These complex modulatory effects were also found to be dose-dependent and increased with more rMSCs in coculture. It was revealed that the paracrine trophic factors secreted by rMSCs, potentially including FGF-1, VEGF-A and PDGFbb, mediated the compounding effects and the RhoA/ROCK signaling pathway was also involved.

The most pronounced observation was the transition of rACs from a rounded or polygonal morphology to an elongated, fibroblast-like shape in coculture with rMSCs. Such a morphological change resembled that observed during chondrocyte dedifferentiation upon serial monolayer culture, wherein a rounded morphology is correlated with the differentiated phenotype of chondrocytes^28^,^29^. Cell shape is generally linked to cellular differentiation and often mediated through RhoA/ROCK signaling that ultimately regulates myosin-generated cytoskeletal tension, and a higher ROCK activity is associated with cell spreading^27^,^30^. In coculture, when a ROCK inhibitor Y27632 was added, the change in cell shape was abolished, confirming the potential involvement of RhoA/ROCK signaling. Y27632 has been shown to prevent dedifferentiation of human ACs by retaining a rounded morphology, upregulating chondrocytic marker genes (*Sox9, Col2a1* and *Acan*) and stimulating cell proliferation^31^. Also, expression of the master transcription factor of chondrogenesis, Sox9, was found to be regulated by RhoA/ROCK signaling and actin polymerization, possibly involving protein kinase A activity^32^,^33^. In consistent with these, in the present study, addition of Y27632 in coculture led to a greater production of cartilaginous ECM (type II collagen and GAG) and upregulation of chondrocytic genes (*Acan, Col2a1 and Sox9*), albeit being lower in coculture at high densities of rMSCs than those in monoculture. However, proliferation of rACs in coculture was not affected by Y27632. In association with the shape transition of rACs, gene expression of integrins was found to be perturbed by rMSCs, with most of integrins upregulated upon coculture, and addition of Y27632 reversed this change. Additionally, expression of other ECM components including *Col1a1* and *Fn1* was also affected by coculture. This together might have a significant impact on applying MSCs and ACs in combination to cartilage regeneration, since the integrin profile of ACs has been tightly linked to both developmental and pathological conditions of cartilage, implicating active cellular responses to the evolving microenvironments^34^,^35^. Along with the change in the integrin profile, expression of *ROCK1* and *ROCK2* was also shown to be upregulated in coculture, which was inhibited by addition of Y27632. This is in line with the existence of a feedback loop in the regulation of cell shape, cell adhesion (through integrins), cytoskeleton tension and ROCK signaling^27^, further indicating the drastic perturbation of rACs by rMSCs in coculture.

Proliferation stimulation of rACs by rMSCs was noted in coculture and the number of rACs within 6 days at the presence of the highest density of rMSCs was almost twice as that in monoculture. MSCs are known to secrete a plethora of trophic factors including FGF, VEGF and PDGF^36^,^37^,^38^. In the present study, by adding an inhibitor of VEGFR1/2/3, FGFR1/2/3 and PDGFRα/β, BIBF1120, proliferation of rACs was drastically inhibited in coculture. At the same time, cell morphology, GAG production and gene expression in coculture were all reverted to those as in monoculture, implicating the critical roles of these growth factors in mediating interactions between rMSCs and rACs. This observation was in contrast to a study by Wu *et al*., wherein MSCs stimulated both proliferation and GAG production of chondrocytes in a pellet coculture with cell-cell contacts^21^,^39^. However, in our previous study, while MSCs had been shown to downregulate both cartilaginous ECM production and gene expression of ACs encapsulated in alginate gel beads in non-contact coculture, stimulation of proliferation was not observed^26^. Moreover, Lee *et al*. reported that adipose-derived MSCs could downregulate the differentiated phenotype of costochondral chondrocytes by secreting VEGF-A and FGF-2 in a similar coculture setting and a reduction in chondrocyte proliferation in coculture was noted^25^. This was possibly due to variations in the secretome of MSCs derived from different tissue origins (bone marrow versus adipose tissue). To further confirm our finding, FGF-1, VEGF-A and PDGFbb were supplemented in individual or in combination in monoculture of rACs. Growth factors including TGF-β1, FGF-2 and PDGFbb had previously been applied to stimulate proliferation of ACs during monolayer expansion^40^. It should be pointed out that the three tested growth factors (FGF-1, VEGF-A and PDGFbb) exerted differential effects on rACs. FGF-1, PDGFbb and FGF-1&VEGF-A&PDGFbb, but not VEGF-A or FGF-1&VEGF-A, promoted proliferation of rACs. These growth factors also displayed distinct effects on morphology, gene expression and GAG production of rACs. In fact, Lee *et al*. suggested the opposite effects of VEGF-A and FGF-2 on proliferation of costochondral chondrocytes, with VEGF-A showing the inhibitory effect^25^. Previously, Wu *et al*. reported that MSCs provoked proliferation of chondrocytes in a pellet coculture possibly through FGF-1 signaling^41^. In the present study, when a specific FGFR-1 inhibitor (PD173046) was added to coculture, changes in both proliferation and morphology of rACs induced by rMSCs were inhibited, confirming the significant role of FGF signaling. However, taken into consideration of all aspects including the morphology, GAG production, gene expression as well as proliferation of rACs, only a combination of FGF-1, VEGF-A and PDGFbb (FGF-1&VEGF-A&PDGFbb) could essentially recapitulate the compounding effects of rMSCs on rACs.

Coculture between MSCs and ACs has been studied in diverse settings with or without cell-cell contacts and in growth or chondrogenic induction medium (i.e., with TGF-β supplemented), which possibly has caused the inconsistency in interpreting the interactions across different studies^6^. It is challenging in analyzing a mixed cell population in coculture with physical contact, generally requiring tedious molecular technologies and thus generating vague observations^21^,^42^. Nevertheless, initial attention has been attracted to uncovering the influence of ACs exerted on MSCs and it is claimed that the paracrine secretion by ACs (e.g., parathyroid hormone-related protein) and/or direct cell-cell contacts (e.g., forming gap junctions) are responsible for improved chondrogenesis of MSCs^12^,^13^,^14^,^15^,^24^,^43^,^44^. However, bidirectional interactions between MSCs and ACs are recently also appreciated^23^,^24^. In some reports, MSCs in coculture did not undergo chondrogenesis at all and increased chondrocyte proliferation and matrix production were considered to be the major contributing factors in tissue formation^20^,^21^. Besides the debate on the effects of coculture between MSCs and ACs, the molecular mechanisms mediating the reciprocal interactions are still far from being elaborated^6^. Culture medium (with or without chondrogenic factors) can modulate the secretome of cells and complicate the observations in coculture^25^,^45^. Lai *et al*. demonstrated that the interactions between osteoarthritic (OA) chondrocytes and adipose-derived MSCs were dependent on both cell proximity and TGF-β3 induction^46^. In a pellet coculture with cell-cell contacts in serum-containing growth medium, FGF-1 was demonstrated to be the candidate factor secreted by MSCs that stimulated chondrocyte proliferation^41^. In a non-contact coculture in chondrogenic medium (TGF-β1 supplemented), Acharya *et al*. found no paracrine interactions between human MSCs and human ACs^24^. Moreover, Manferdini *et al*. found that the anti-inflammatory effect of MSCs on OA chondrocytes depended on the inflammatory status of chondrocytes^47^. In the present study, in the non-contact coculture in growth medium, growth factors, potentially including FGF-1, VEGF-A and PDGFbb, were exclusively demonstrated to antagonistically and synergistically mediate the paracrine effects of rMSCs that downregulated the differentiated phenotype of rACs and perturbed the expression of a large array of genes^25^,^36^,^48^. It should be noted that serum-containing growth medium was applied in the present study. Since serum composition is intrinsically undefined, a future study in chemically defined medium is warranted to characterize unequivocally the effects of the secretome of MSCs on ACs.

Based on the above analyses, a potential molecular mechanism in mediating the modulatory effects of MSCs on ACs in coculture can be proposed. In literature, crosstalk between growth factors and RhoA/ROCK is implicated, leading to the association between cell shape and growth factor signaling^49^. In addition, there is a feedback loop in cell shape, adhesion (via integrins), cytoskeleton tension and ROCK signaling and chondrocyte differentiation is strongly regulated by actin cytoskeleton and cell adhesion^27^,^35^. Moreover, integrin signals can work in concert with the signaling of growth factors that converge on mitogen activated protein kinase/extracellular signal-regulated kinases (MAPK/ERK) pathway^35^. As schematically illustrated in Fig. 7, MSCs secrete a variety of trophic factors potentially including FGF-1, VEGF-A and PDGFbb; these paracrine factors transduce intracellular signaling, possibly engaging both MAPK/ERK and RhoA/ROCK pathways; these pathways eventually regulate gene expression, inducing shape change, provoking cell proliferation and downregulating chondrocytic gene expression and cartilaginous ECM production.

One of the challenges in applying ACs for cartilage regeneration is dedifferentiation and coculture between MSCs and ACs has been expected to overcome this issue^5^. Dedifferentiated chondrocytes display a fibroblast-like morphology, reduced ECM synthesis, downregulated chondrocytic gene expression (e.g. *Col2a1*) and upregulated fibroblastic gene expression (e.g. *Col1a1*) as well as an altered cell surface antigen profile^2^,^50^. In coculture with rMSCs, by displaying a fibroblast-like shape, downregulated chondrocytic gene expression (*Acan, Col2a1* and *Sox9*), upregulated fibroblastic gene expression (*Vcan* and *Col1a1*), and reduced cartilaginous ECM production (GAG and type II collagen), rACs seemed to undergo an expedited dedifferentiation process compared to those in control monoculture. In literature, to evaluate the differentiated phenotype of chondrocytes, gene expression ratios of *Col2a1*/*Col1a1* and *Acan*/*Vcan* are considered as convenient quantitative indices^48^,^51^ and recently, a high ratio of *CD14*/*CD90* (i.e., *Thy1*) has also been correlated with human chondrocyte differentiation^50^,^52^. We had previously confirmed that dedifferentiation of rACs was associated with upregulation of *CD90* and a steady level of *CD14* expression^26^. Strikingly, upon coculture with rMSCs, upregulation of *CD14* and downregulation of *CD90* were consistently observed, suggesting that coculturing rMSCs with rACs might have not provoked a *bona fide* chondrocyte dedifferentiation process. Monolayer culture of ACs has been speculated to be a selective expansion of progenitor cells^52^. However, it seemed not to be the case in coculture in the present study, since gene expression of *CD90*, a well-known surface molecular marker for MSCs, was downregulated in rACs upon coculture. Nevertheless, considering that dedifferentiated chondrocytes might share a similar pattern of surface markers with those OA chondrocytes^52^, changes of rACs in coculture with rMSCs distinct from dedifferentiation might be considered beneficial when applying these cells in cartilage repair. As a matter of fact, ACs expanded in monolayer at the presence of growth factors have been demonstrated to have an improved redifferentiation potential during subsequent chondrogenic induction^40^. In this sense, chondrocytes can be conditioned in coculture with MSCs and possibly rejuvenated with a better chondrogenic potential for subsequent cartilage regeneration applications.

Taken together, we have demonstrated that rMSCs could significantly downregulate the differentiated phenotype of rACs in a non-contact coculture setting. The modulatory effects including changes in cell shape, proliferation, gene expression and ECM production can be potentially attributed to soluble factors secreted by rMSCs, including FGF-1, VEGF-A and PDGFbb. Intriguingly, the changes in rACs induced by coculture are not replicating those of chondrocyte dedifferentiation, especially according to the gene expression profile of surface molecules (i.e., *CD90* and *CD14*). These findings may be of great significance to applying cell-based strategies for achieving cartilage regeneration.

## Methods

### Cell isolation

Both AC and MSCs were isolated from rabbits as previously described^26^,^53^,^54^. All animal experiments were performed in accordance with the guidelines for the care and use of laboratory animals at Shanghai Laboratory Animal Center (Shanghai) and the protocols were approved by the institutional animal care and use committee of Shanghai Laboratory Animal Center. Briefly, cartilage slices were taken from the patellofemoral groove and femoral condyles of 2-month-old New Zealand white rabbits and digested in 0.25% trypsin/EDTA (Gibco, Grand Island, NY, USA) in brief followed by 0.1% collagenase II (200 units/mg; Invitrogen, Grand Island, NY, USA) for about 5 h on a shaker at 37 °C and 5% CO_2_ in an incubator. Released cells were collected by centrifugation for 10 minutes at 2000 rpm and rinsed with phosphate buffered saline (PBS) containing 100 U/ml penicillin and 100 U/ml streptomycin. Rabbit ACs (rACs) were then either cultured in chondrocyte growth medium consisting of high-glucose Dulbecco’s modified Eagle’s medium (DMEM, Gibco) supplemented with 10% fetal bovine serum (FBS; Hyclone, Logan, UT, USA), 0.1 mM nonessential amino acids, 0.4 mM proline, 0.05 mg/mL vitamin C, 100 U/mL penicillin and 100 U/mL streptomycin or frozen down for future use.

To isolate MSCs, bone marrow harvested from rabbit femur and tibia was resuspended in PBS and gently layered on an equal volume of Ficoll-Paque Plus solution (density: 1.077 g/mL; GE Healthcare, Pittsburgh, PA. USA) in a 50 mL centrifuge tube. Following centrifugation at 400 g for 30 min, low-density mononuclear cells located in the top second layer was collected and rinsed with PBS. Cells were then plated at 1 × 10^5^ cells/cm^2^ in growth medium consisting of α-Minimum Essential Medium (α-MEM; Gibco) supplemented with 10% FBS, 100 units/mL of penicillin and 100 units/mL of streptomycin in a humidified atmosphere of 5% CO_2_ at 37 °C and subcultured at a density of 5 × 10^3^ cells/cm^2^. Rabbit MSCs (rMSCs) were validated to be CD29^+^, CD44^+^, CD14^−^ and CD45^−^ and demonstrated the capability of differentiation into chondrocytes, osteoblasts and adipocytes.

### Coculture studies

**Figure 1:**
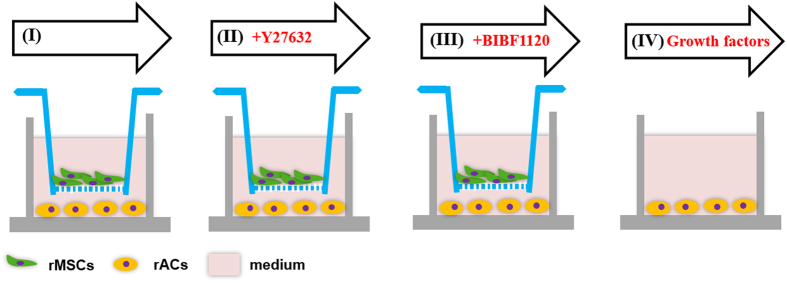
Coculture design. In a typical coculture setup (**I**), rACs were first seeded in 12-well tissue culture plates in chondrocyte growth medium. Then, a Transwell insert was placed in each well and rMSCs were seeded in the inserts to initiate coculture. Y27632 (**II**) or BIBF1120 (**III**) was optionally supplemented in growth medium in coculture. In addition, a monoculture of rACs was also set up and the medium was supplemented with growth factors (I**V**).

**Figure 2:**
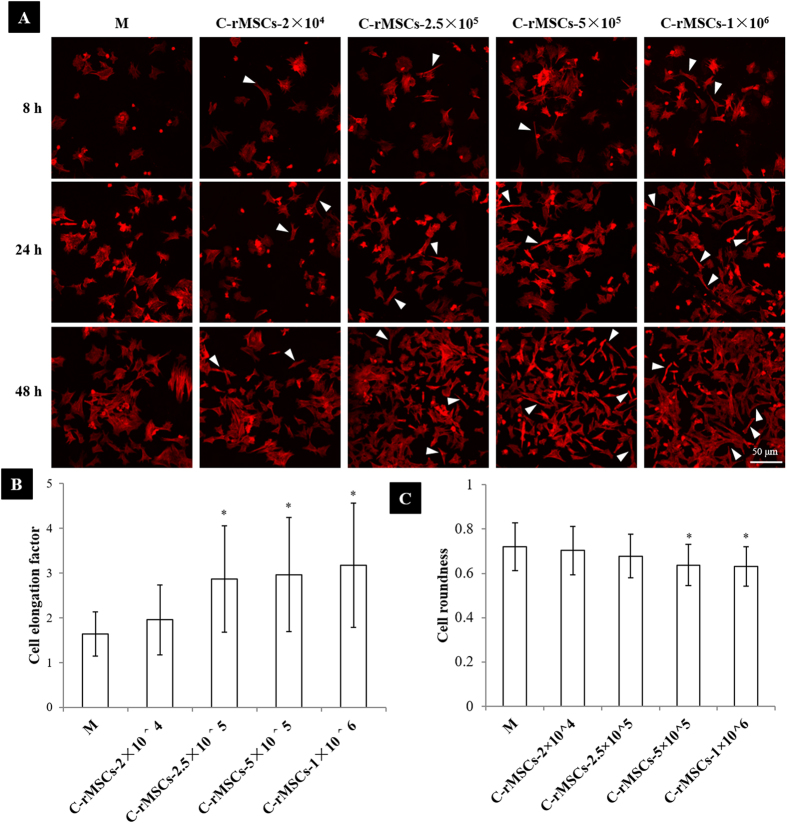
rMSCs induced a morphological transformation of rACs in coculture. (**A**) F-actin staining (8 h, 24 h and 48 h), (**B**) cell elongation factor (48 h) and (**C**) cell roundness (48 h). **M**: rACs monoculture as control; **C-rMSCs−2 × 10**^**4**^, **−2.5 × 10**^**5**^, **−5 × 10**^**5**^ and **−1 × 10**^**6**^: rACs cocultured with rMSCs at 2 × 10^4^, 2.5 × 10^5^, 5 × 10^5^ and 1 × 10^6^ cells/well, respectively; *p < 0.05; n > 30.

**Figure 3:**
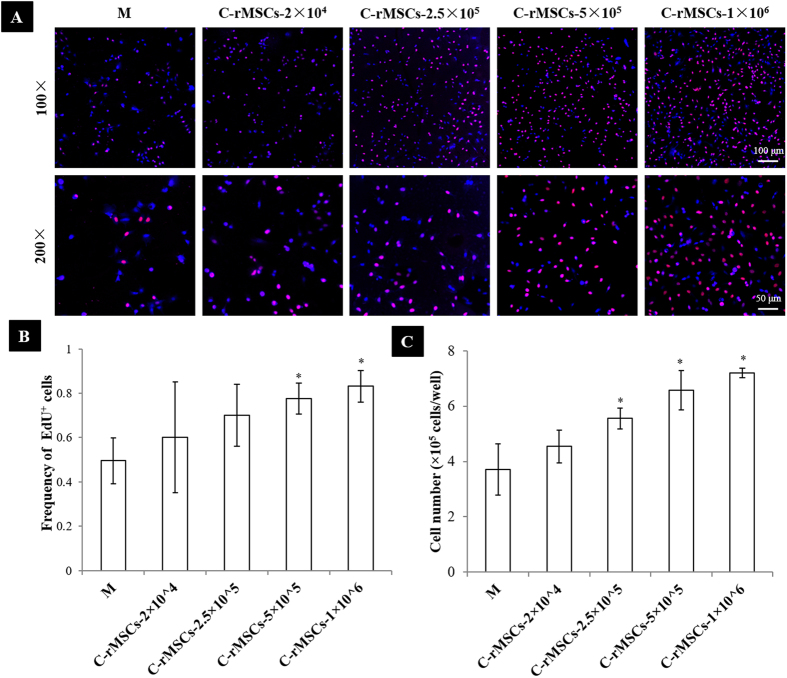
rMSCs stimulated proliferation of rACs in coculture. (**A**) EdU staining and (**B**) frequency of EdU^+^ cells (24 h), and (**C**) cell number (day 6). **M**: rACs monoculture as control; **C-rMSCs−2 × 10**^**4**^, **−2.5 × 10**^**5**^, **−5 × 10**^**5**^ and **−1 × 10**^**6**^: rACs cocultured to rMSCs at 2 × 10^4^, 2.5 × 10^5^, 5 × 10^5^ and 1 × 10^6^ cells/well, respectively; *p < 0.05; n = 3.

**Figure 4:**
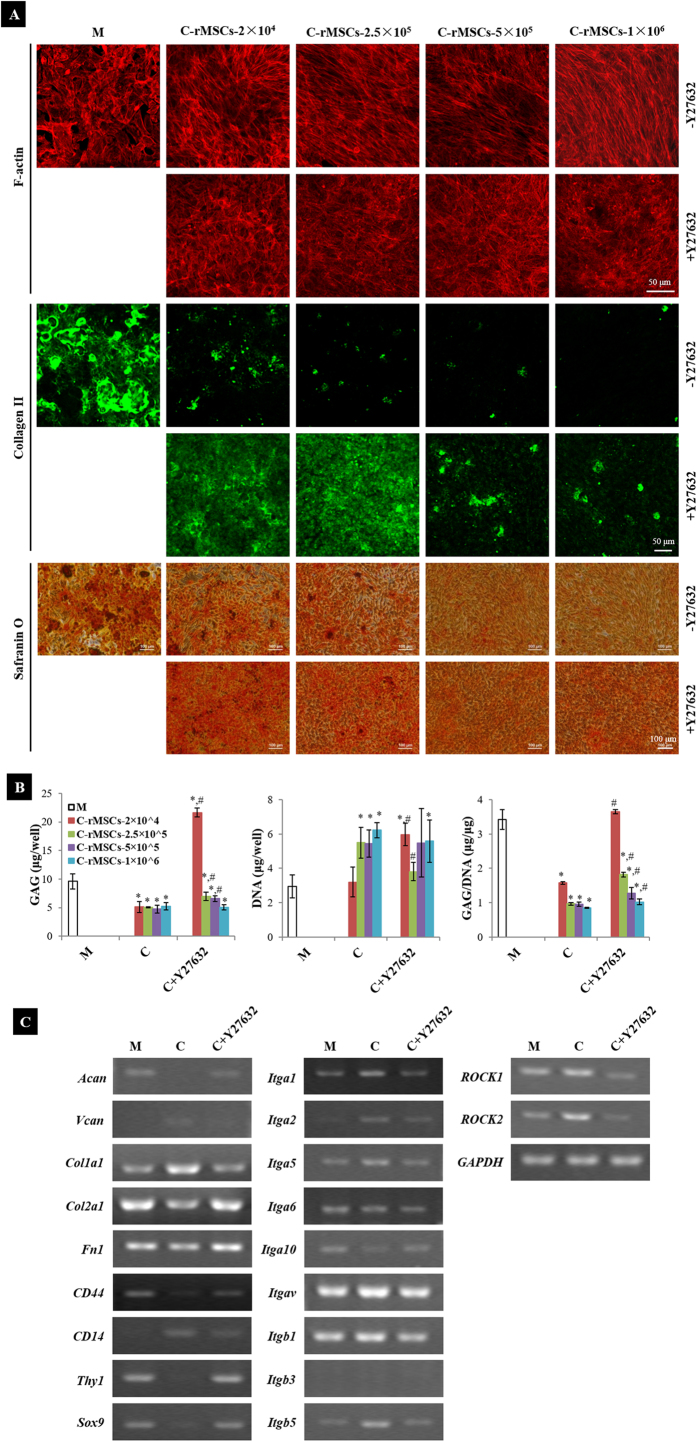
Y27632 inhibited the effects of rMSCs on rACs. (**A**) F-actin staining (day 4), immunofluorescence staining for type II collagen and safranin O staining for GAG (day 6), (**B**) quantification of DNA and GAG (day 6) and (**C**) gene expression (day 4). **C**: coculture; **M**: rACs monoculture as control; **C-rMSCs−2 × 10**^**4**^, **−2.5 × 10**^**5**^, **−5 × 10**^**5**^ and **−1 × 10**^**6**^: rACs cocultured with rMSCs at 2 × 10^4^, 2.5 × 10^5^, 5 × 10^5^ and 1 × 10^6^ cells/well, respectively; **C + Y27632**: coculture with Y27632 supplemented;** + Y27632**: Y27632 supplemented; **-Y27632**: no Y27632 supplemented; *p < 0.05, compared to **M**; #p < 0.05, compared to **C**; n = 3.

**Figure 5:**
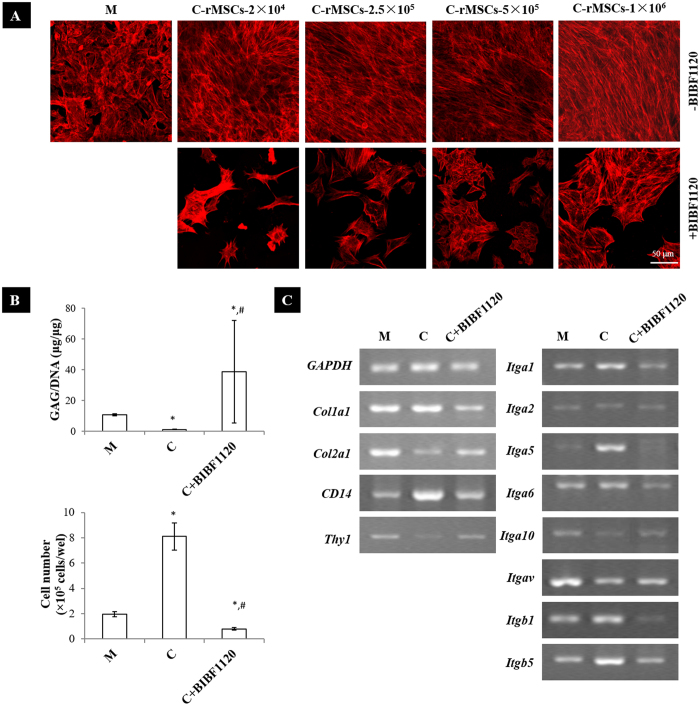
BIBF1120 reversed the effects of rMSCs on rACs. (**A**) F-actin staining (day 4), (**B**) quantification of GAG and cell number (day 6) and (**C**) gene expression (day 4). **C**: coculture; **M**: rACs monoculture as control; **C−rMSCs−2 × 10**^**4**^, **−2.5 × 10**^**5**^, **−5 × 10**^**5**^ and **−1 × 10**^**6**^: rACs cocultured with rMSCs at 2 × 10^4^, 2.5 × 10^5^, 5 × 10^5^ and 1 × 10^6^ cells/well, respectively; **C + BIBF1120**: coculture with BIBF1120 supplemented; **+BIBF1120**: BIBF1120 supplemented; **−BIBF1120**: no BIBF1120 supplemented; *p < 0.05, compared to **M**; #p < 0.05, compared to **C**; n = 3.

**Figure 6:**
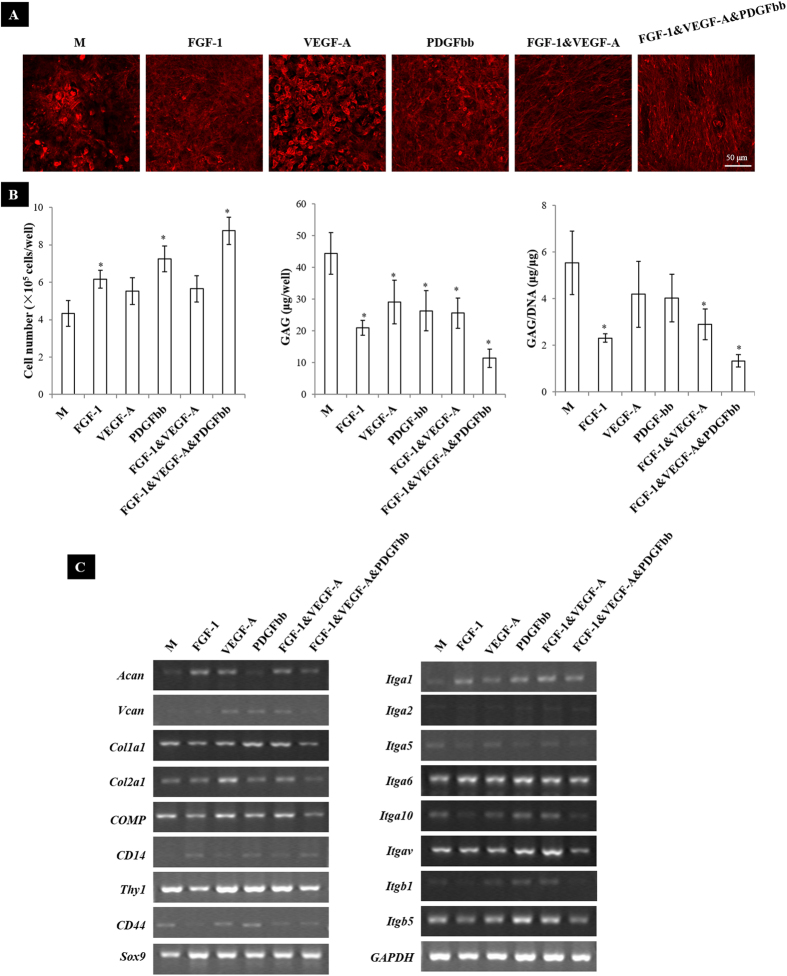
FGF-1, VEGF-A and PDGFbb partially mimicked the effects of rMSCs on rACs. (**A**) F-actin staining (day 6), (**B**) quantification of GAG and cell number (day 6) and (**C**) gene expression (day 4). **M**: rACs monoculture without growth factors supplemented as control; *p < 0.05, compared to **M**; n = 3.

**Figure 7:**
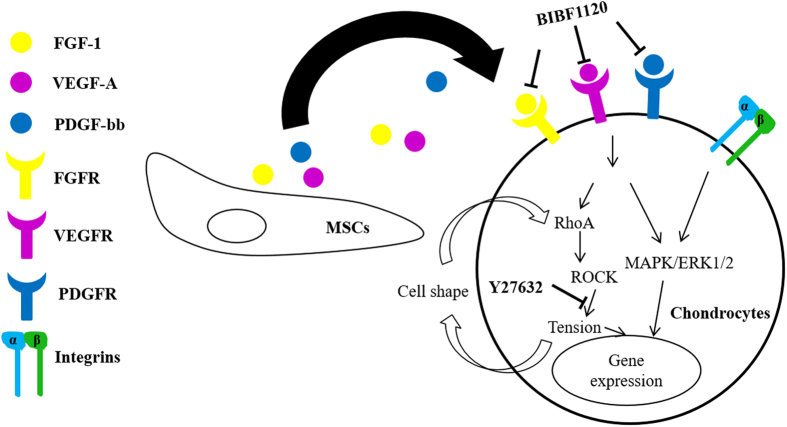
A proposed molecular mechanism for the modulatory effects of rMSCs on rACs.

In a typical experimental setup as illustrated in Fig. 1, rACs (passage 1, P1) were first seeded in 12-well tissue culture plates at a density of 5 × 10^3^ cells/cm^2^ in chondrocyte growth medium (2 mL/well). After 1 day, a Transwell insert (pore size 0.4 μm; Millipore, Billerica, MA, USA) was placed in each well and rMSCs (P3) were seeded in the inserts at four different densities (2 × 10^4^, 2.5 × 10^5^, 5 × 10^5^ and 1 × 10^6^ cells/well) to initiate coculture. The culture lasted for 6 days and medium was refreshed every other day. In some coculture experiments (Fig. 1), either the ROCK inhibitor Y27632 (10 μM, Selleck Chemicals, Houston, TX, USA) or BIBF1120 (1.08 μM, Selleck Chemicals), an inhibitor of vascular endothelial growth factor receptor-1/2/3 (VEGFR1/2/3), fibroblast growth factor receptor-1/2/3 (FGFR1/2/3) and platelet-derived growth factor receptor-α/β (PDGFRα/β) receptors was supplemented in the medium at the initiation of coculture.

In some other experiments (Fig. 1), rACs (P1) were plated at 5 × 10^3^ cells/cm^2^ in 12-well plates in chondrocyte growth medium and after 1 day, the medium was supplemented with either FGF-1 (5 ng/mL; recombinant human FGF acidic, R&D Systems, Minneapolis, MN, USA), VEGF-A (10 ng/mL; recombinant human VEGF_165_, R&D Systems), PDGFbb (10 ng/mL; recombinant human PDGF-BB, R&D Systems), FGF-1 (5 ng/mL) & VEGF-A (10 ng/ml) or FGF-1 (5 ng/mL) & VEGF-A (10 ng/ml) & PDGFbb (10 ng/mL). Culture lasted for 6 days and medium containing growth factors was refreshed every other day.

### F-actin staining

Cultured rACs in plate wells were rinsed with PBS, fixed with 4% paraformaldehyde in PBS and permeated with 0.1% Triton X-100 in PBS. After rinse with PBS, samples were treated with Rhodamine-phalloidin (5 μg/mL; Invitrogen) in PBS containing 1% bovine serum albumin (BSA, Sigma, St. Louis, MO, USA) in dark for 20 min. Fluorescence images were acquired using confocal laser scanning microscopy (CLSM, TI-LU4SU, Nikon, Tokyo, Japan). Cell elongation factor, defined as the long axis/ short axis, and R_c_ = 2(πA)^1/2^/L describing cell roundness^55^, were determined based on the image analysis using Image J software. For each group, 2 images (400x magnification; >30 cells in total) were analyzed.

### Histochemical and immunofluorescence staining

Safranin O staining was performed to detect the production of GAG by rACs. Cells in plate wells were fixed in 4% paraformaldehyde for 15 min, rinsed with PBS twice and incubated with 0.1% safranin O solution in distilled water (Sigma) for 30 min. Samples were dehydrated with an ethanol series and mounted using resinous medium for visualization under a phase contrast microscopy.

For immunofluorescence detection of type II collagen, cells were fixed in 4% paraformaldehyde and treated with 0.25% Triton X-100 in PBS (PBT), followed by a blocking buffer (1% BSA in PBT). Samples were then incubated with mouse anti-rabbit type II collagen antibody (Millipore) at a dilution of 1:100 and further detected using. Specific antibody binding was detected by incubation with fluorescein isothiocyanate-conjugated goat anti-mouse secondary antibody (1:200 dilution; Invitrogen) at room temperature for 2 h. Cell nuclei were counterstained with 4′, 6-diamidino-2-phenylindole (DAPI) and fluorescent images were obtained on a fluorescence microscope (Eclipse Ti-S, Nikon).

### EdU assay

Cell proliferation was analyzed by using the Cell-Light^TM^ EdU kit (Guangzhou RiboBio Co., Ltd, Guangzhou, China). 5-Ethynyl-2′-deoxyuridine (EdU) is a thymidine nucleoside analogue and it can replace thymidine to be incorporated into DNA during proliferation. To evaluate the proliferation of rACs, coculture was set up as above described. However, after 1 day of coculture initiated, EdU (10 μM) was added to the medium and incubated for 4 h. The culture was terminated and cells were fixed in 4% paraformaldehyde. Detection of incorporated EdU was conducted according the manufacturer’s instruction. Cell nuclei were counterstained with DAPI and fluorescent images were acquired under CLSM. For each group, three images were analyzed to determine the fraction of EdU^+^ cells.

### Biochemical assays

Cells in each well were treated with 200 μL papain (16–40 U/mg, Sigma) solution (125 μg/mL papain, 5 mM L-cysteine, 100 mM Na_2_HPO_4_, and 5 mM EDTA, pH 6.2) at 60 °C for 16–20 h and the supernatants were used to measure the contents of both DNA and GAG as previously described. DNA content was determined using Hoechst 33258 (Sigma) with calf thymus DNA (Invitrogen) as a standard on a Hoefer DQ300 fluorometer (Hoefer, Holliston, MA, USA). GAG content was determined using 1,9-dimethyl-methylene blue (DMMB)-based spectrometry with chondroitin sulfate (from shark cartilage, Sigma) as a standard. The absorption at 525 nm was recorded on a DU^®^ 730 UV/Vis spectrophotometer (Beckman Coulter, Lawrence, KS, USA). GAG content was normalized to DNA content and expressed as GAG/DNA (μg/μg). All biochemical assays were performed in triplicates.

### Reverse transcriptase-polymerase chain reaction (RT-PCR)

To isolate total RNA, rACs were lyzed with Trizol (Invitrogen) and RNA extraction was performed according to the manufacturer’s instruction. cDNA was synthesized from 1 μg of mRNA with M-MLV Reverse Transcriptase (Promega, Madison, WI, USA) using oligo(dT) and amplified using PCR Master Mix (TaKaRa, Kusatsu, Shiga, Japan) on a thermal cycler (TC-XP-A, Bioer, Hangzhou, China). PCR products were analyzed by using 2% agarose gel electrophoresis. *GAPDH* was used as housekeeping gene. Primers designed for PCR analysis were listed in Table S1.

### Statistical analysis

Values were presented as average ± standard deviation. Two-tailed, unpaired student’s t-test was applied to determine the significance of difference between values. P < 0.05 were considered statistically significant.

## Additional Information

**How to cite this article**: Xu, L. *et al*. Mesenchymal Stem Cells Reshape and Provoke Proliferation of Articular Chondrocytes by Paracrine Secretion. *Sci. Rep.* **6**, 32705; doi: 10.1038/srep32705 (2016).

## Supplementary Material

Supplementary Information
