# Supplementary material for: Mesenchymal Stem Cells Reshape and Provoke Proliferation of Articular Chondrocytes by Paracrine Secretion
Source: Sci Rep. 2016 Sep 6;6:32705. doi: 10.1038/srep32705 (PMC5011711; doi:10.1038/srep32705)
Supplement: Supplementary Information [file srep32705-s1.pdf]

Mesenchymal Stem Cells Reshape and Provoke Proliferation of Articular Chondrocytes by Paracrine Secretion

Lei Xu, Yuxi Wu, Zhimiao Xiong, Yan Zhou, Zhaoyang Ye*, Wen-Song Tan*

State Key Laboratory of Bioreactor Engineering, East China University of Science and Technology, Shanghai 200237, China

Running title: MSCs Reshape and Provoke Proliferation of Chondrocytes

All authors would like to indicate that there is no conflict of interest with regard to the work.

* To whom correspondence should be addressed:

State Key Laboratory of Bioreactor Engineering

East China University of Science and Technology

130 Mei-Long Road, P. O. Box 309#

Shanghai 200237, P. R. China

Tel: +86-21-64251570

Fax: +86-21-64252099

E-mail: zhaoyangye@ecust.edu.cn (Zhaoyang Ye)

wstan@ecust.edu.cn (Wen-Song Tan)

Supplementary Methods:

Preparation of rMSCs-conditioned medium

P3 rMSCs were plated at 5×10^3 cells/cm^2 in T150 flasks (TPP) in growth medium. Once reaching 100% confluence, the medium was discarded and cells were rinsed once with PBS. Then, α-MEM without serum (40 mL/flask) was added and incubated for 2 days. The medium was collected, supplemented with FBS to 10% and mixed with chondrocyte growth medium at a 1:2 volume ratio, which was defined as conditioned medium (CM).

Coculture with an FGFR1 inhibitor supplemented

Coculture was set up as described by seeding P1 rACs at 5×10^3 cells/cm^2 in chondrocyte growth medium. After 1 day, P3 rMSCs were seeded in the Transwell inserts at four different densities (2×10^4, 2.5×10^5, 5×10^5 and 1×10^6 cells/well) to initiate coculture and at the same time, the FGFR1 inhibitor PD173074 (110 nM, Selleck) was add to the medium. Culture lasted for 6 days and medium containing the inhibitor was refreshed every other day.

Monoculture of rACs in serum-free medium supplemented with growth factors

rACs (P1) were plated at 5×10^3 cells/cm^2 in 12-well plates in chondrocyte growth medium and after 1 day, the medium was changed to serum-free medium consisting of DMEM supplemented with 1% ITS (0.55 mg/mL insulin, 0.55 mg/mL transferrin, 0.00067 mg/mL sodium selenite, 11 mg/mL sodium pyruvate; Gibco), 1.25 mg/mL BSA, 100 U/mL penicillin and 100 U/mL streptomycin, which was further supplemented with either FGF-1 (5 ng/mL),

VEGF-A (10 ng/mL), PDGFbb (10 ng/mL), FGF-1 (5 ng/mL) & VEGF-A (10 ng/ml) or FGF-1 (5 ng/mL) & VEGF-A (10 ng/ml) & PDGFbb (10 ng/mL). Culture lasted for 6 days and medium containing growth factors was refreshed every other day.

Immunofluorescence staining

For immunofluorescence staining of α-tubulin and vimentin, 1:100 dilution of rabbit anti-rabbit α-tubulin antibody (Abcam) and 1:500 dilution of rabbit anti-rabbit vimentin antibody (Abcam) were used, respectively.

Scanning electron microscopy (SEM)

In coculture, rACs were plated on glass coverslips. After coculture, cells were fixed with 2.5% glutaraldehyde overnight, dehydrated using a series of concentrations of ethanol (50%, 70%, 90% and 100%), and then air-dried. Samples were then were staged on the stub, sputter-coated with gold and then examined by using SEM (Hitachi S-3400N, Japan).

Table S1. The Primers used for RT-PCR analysis.

Gene	Forward and Reverse primers (5’→3’)	Product size (bp)
GAPDH	5’-TCACCATCTTCCAGGAGCGA-3’	293
	5’-CACAATGCCGAAGTGGTCGT-3’	
Col1a1	5’-AAAGGGACACAACGGATTGCAAGG-3’	500
	5’-TCCATAGTGCATCCTTGGTTGGGA-3’	
Col2a1	5'-AGAAGAACTGGTGGAGCAGCAAGA-3'	468
	5'-TTTACAAGAAGCAGACGGGCCCTA-3'	
Acan	5'-TGGAGGTCGTGGTGAAAGG-3'	111
	5'-CAATGATGGCGCTGTTCTGT-3'	
Vcan	5'-TCATCTGGACGGTGACGTGTTTCA-3'	415
	5'-TGGTGTGGTTCTCTTGGGCACTAT-3'	
CD14	5'-ACTGAACATTGCCCAAGCACACAC-3'	387
	5'-TTGCAGCTGAGATCCAGCACATTG-3'	
CD44	5'- CCTGATGTTGATCGCTCCTTAC -3'	410
	5'- CGTGTCTCATCTGCTGTCATAC-3'	
Thy1	5'-TGCTCAGAGACAAGCTGGTCAAGT-3'	423
	5'-CGTCCTGGCTTCCCTTGTCATAAA-3'	
SOX9	5'-TCAAGAAGGAGAGCGAAGAGGACA-3'	342
	5’-ACTTGTAGTCCGGGTGGTCTTTCT-3’	
COMP	5'-CTGGACCCTGAGGGCGAT-3'	110
	5'-CCGTGTACCCCACAGCCAG-3'	
Fn1	5'-CGGACTCAAGTGTTCTGGTTAT-3'	239
	5'-CTGAGGCTGCAGAGTAGTAAAG-3'	
Itga1	5'-CACTCAGAGGAGAACAGATTGG-3'	397
	5'-GTCTGCCCACTTCCATGATAA-3'	
Itga2	5'-CCTTGGATGGCTATGGAGATTTA-3'	479
	5'-GACCTTGGCAGTCTCAGAATAG-3'	
Itga5	5'-GAACAGATGGCCTCCTACTTTG-3'	371
	5'-AGAACCTGGGAAGGCTTAGA-3'	
Itga6	5'-GTGTTTCCCTCCAAGACAGTAG-3'	279
	5'-GCCCTAGGAGTAGCTTTCATTC-3'	
Itga10	5'-GACCTCAGCCTACGTTCAATAC-3'	232
	5'-GACTTGAGACAGTGACAGGAAG-3'	
Itgav	5'-CCGACAGGCACATATTCTACTT-3'	490

	5'-AGGACTTGAGACTCCTCTTATCT-3'	
Itgb1	5'-CCAGAGGTTCCAGAGACATAAAG-3'	281
	5'-CACCCTCCTCATCTCATTCATC-3'	
Itgb3	5'-AGGAGGTGAAGAAGCAGAATG-3'	246
	5'-TGGTGGTAGAGGCAGAGTAA-3'	
Itgb5	5'-GGACTATCCATCCCTTGCTTTAC-3'	224
	5'-GATCCCAGACAGACAGCTCTA-3'	
ROCK1	5'-CTGGTGGTCAGTTGGAGTATTT-3'	278
	5'-CTACAGTGTCTCGGAGTGTTTC-3'	
ROCK2	5'-CGCGAGTGAGAAGGAAGAATTA-3'	385
	5'-GTCTTTACTGTCCAGGGTCATC-3'	

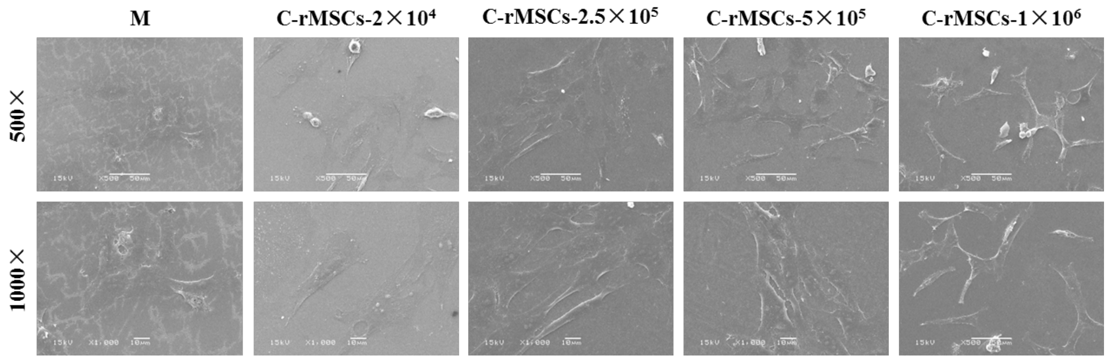

Figure S1. SEM images of rACs upon coculture with rMSCs. rACs were cocultured with different densities of rMSCs for 48 h and then fixed for SEM observation at 500× and 1000× magnifications. Upon coculture, rACs displayed elongated, spindle-like shape, while in control culture (**M**), cells remained a typical polygonal morphology of chodrocytes. **M**: rACs monoculture as control; **C-rMSCs-2×10^{4}**, **-2.5×10^{5}**, **-5×10^{5}** and **-1×10^{6}**: rACs cocultured with rMSCs at 2×10^{4}, 2.5×10^{5}, 5×10^{5} and 1×10^{6} cells/well, respectively.

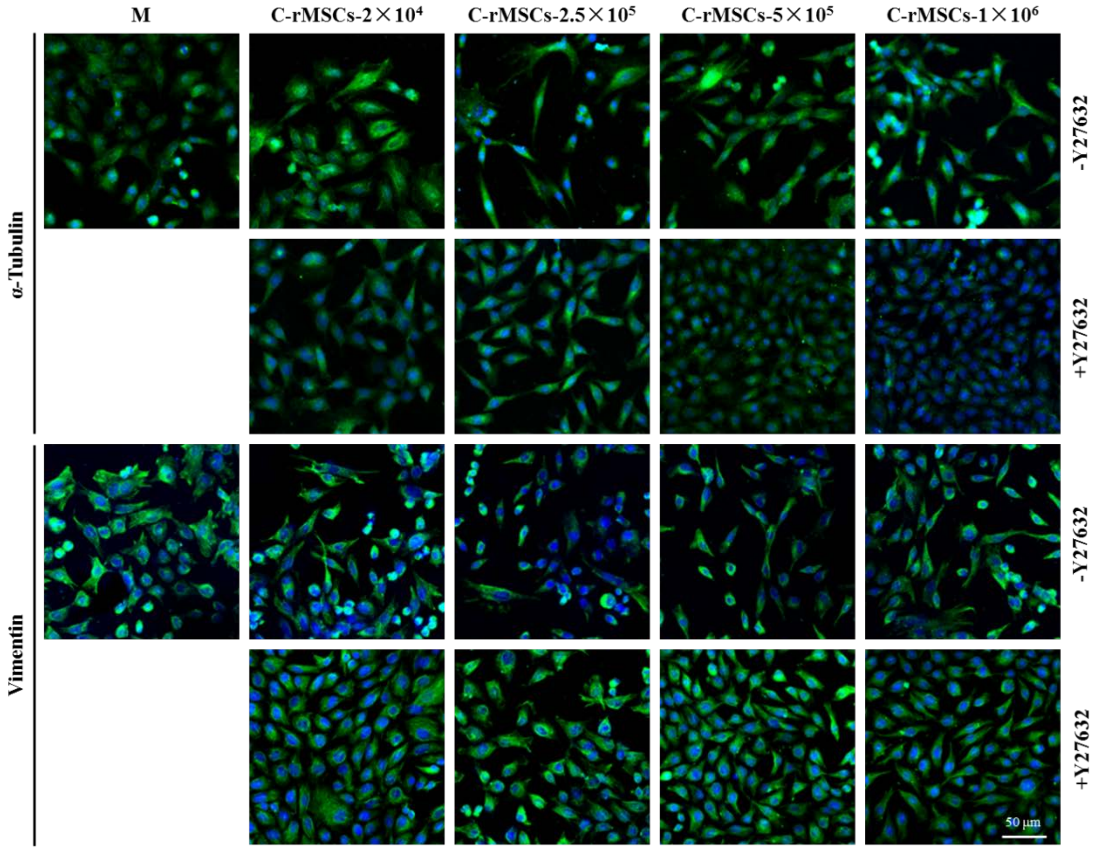

Figure S2. Immunofluorescence staining of α-tubulin and vimentin for rACs in coculture. rACs were cocultured with different densities of rMSCs for 48 h at the presence or absence of Y27632 (10 μM) in the medium. Upon coculture without Y27632, rACs displayed an elongated, spindle-like shape, and in coculture with Y27632 supplemented, cells remained a rounded morphology. **M**: rACs monoculture as control; **C-rMSCs-2×10^{4}**, **-2.5×10^{5}**, **-5×10^{5}** and **-1×10^{6}**: rACs cocultured with rMSCs at 2×10^{4}, 2.5×10^{5}, 5×10^{5} and 1×10^{6} cells/well, respectively; **+Y27632**: Y27632 supplemented; **-Y27632**: no Y27632 supplemented.

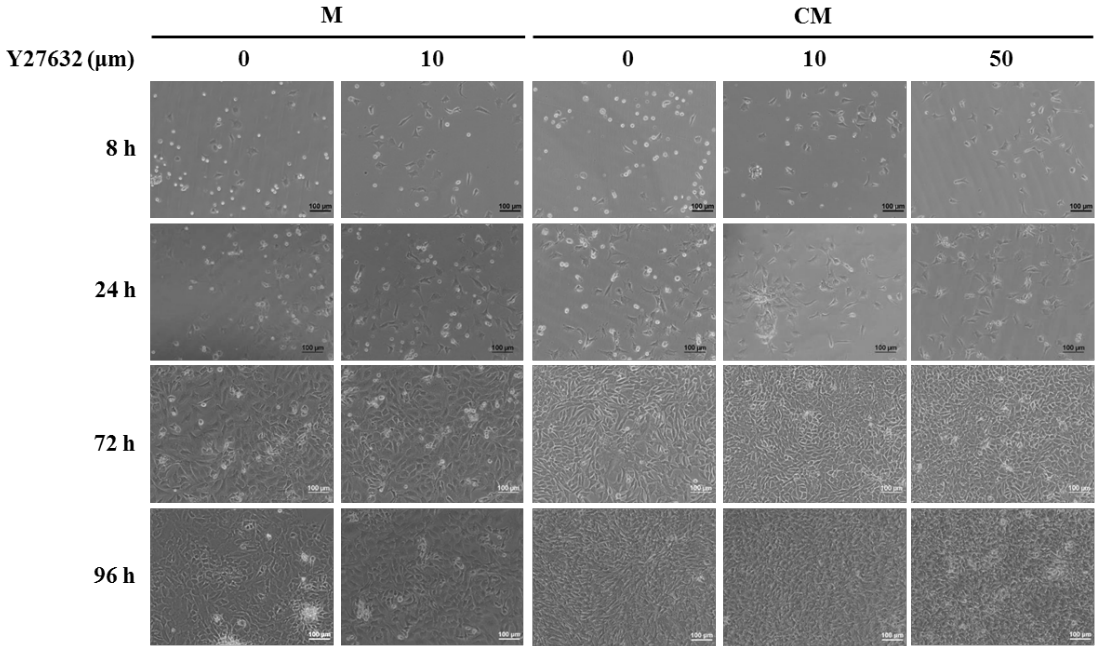

Figure S3. Morphology of rACs in CM with Y27632 supplemented. P1 rACs at 5×10^3 cells/cm^2 in chondrocyte growth medium were seeded and after 1 day, the medium was replaced with CM and cultured for additional 96 h. Different concentrations of Y27632 (0, 10 and 50 μM) were supplemented in media. Under the microscope, rACs turned to spindle-like in CM with time, and the addition of Y27632 in CM inhibited the shage change. In addition, the addition of Y27632 in growth medium had no effect on cell shape. **M**: rACs monoculture in growth medium as controls; **CM**: rACs cultured in CM.

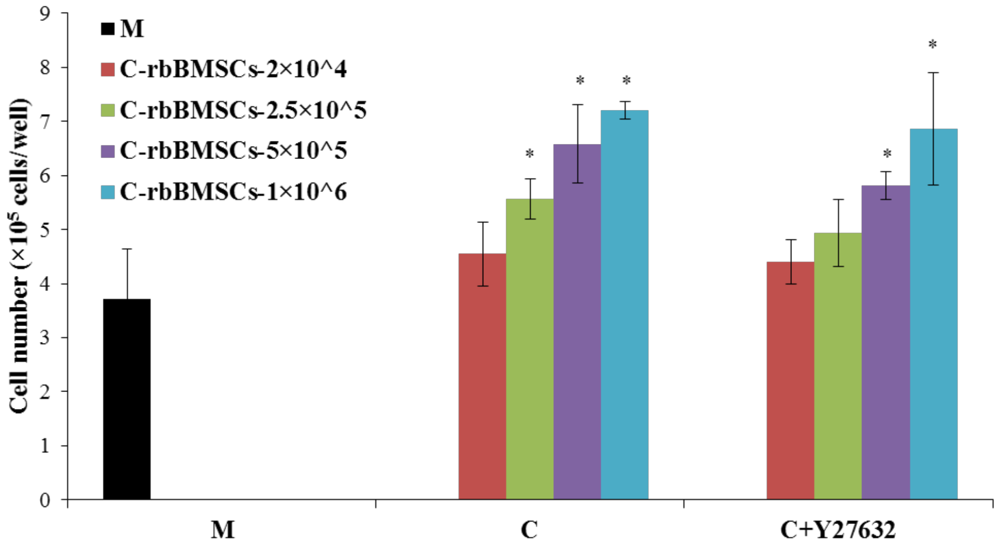

Figure S4. Cell number of rACs in coculture with Y27632 supplemented. Coculture was set up with different densities of rMSCs with Y27632 (10 μM) supplemented or not, and lasted for 6 days. In coculture, the number of rACs increased with an increasing density of rMSCs. However, no significant changes were detected with the addition of Y27632. **M**: rACs monoculture as control; **C-rMSCs-2×10^4**, **-2.5×10^5**, **-5×10^5** and **-1×10^6**: rACs cocultured with rMSCs at 2×10^4, 2.5×10^5, 5×10^5 and 1×10^6 cells/well, respectively; **C+Y27632**: Y27632 supplemented in coculture; **C**: no Y27632 supplemented in coculture; *: $p<0.05$, compared with **M**; n=3.

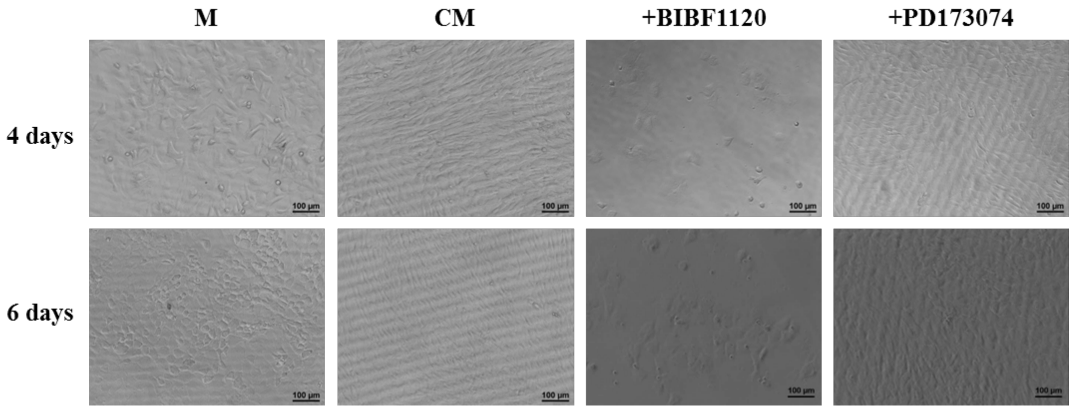

Figure S5. Morphology of rACs in CM with BIBF1120 or PD173074 supplemented. P1 rACs at 5×10^3 cells/cm^2 in chondrocyte growth medium were seeded and after 1 day, the medium was replaced with CM and cultured for additional 6 days. BIBF1120 (1.08 μM) or PD173074 (110 nM) were supplemented in the medium. Under the microscope, rACs turned to spindle-like in CM on day 4 and 6, and the addition of BIBF1120 or PD173074 in CM inhibited the shage change. **M**: rACs monoculture in growth medium as controls; **CM**: rACs cultured in CM; **+BIBF1120** and **+PD173074**: rACs cultured in CM supplemented with BIBF1120 and PD173074, respectively.

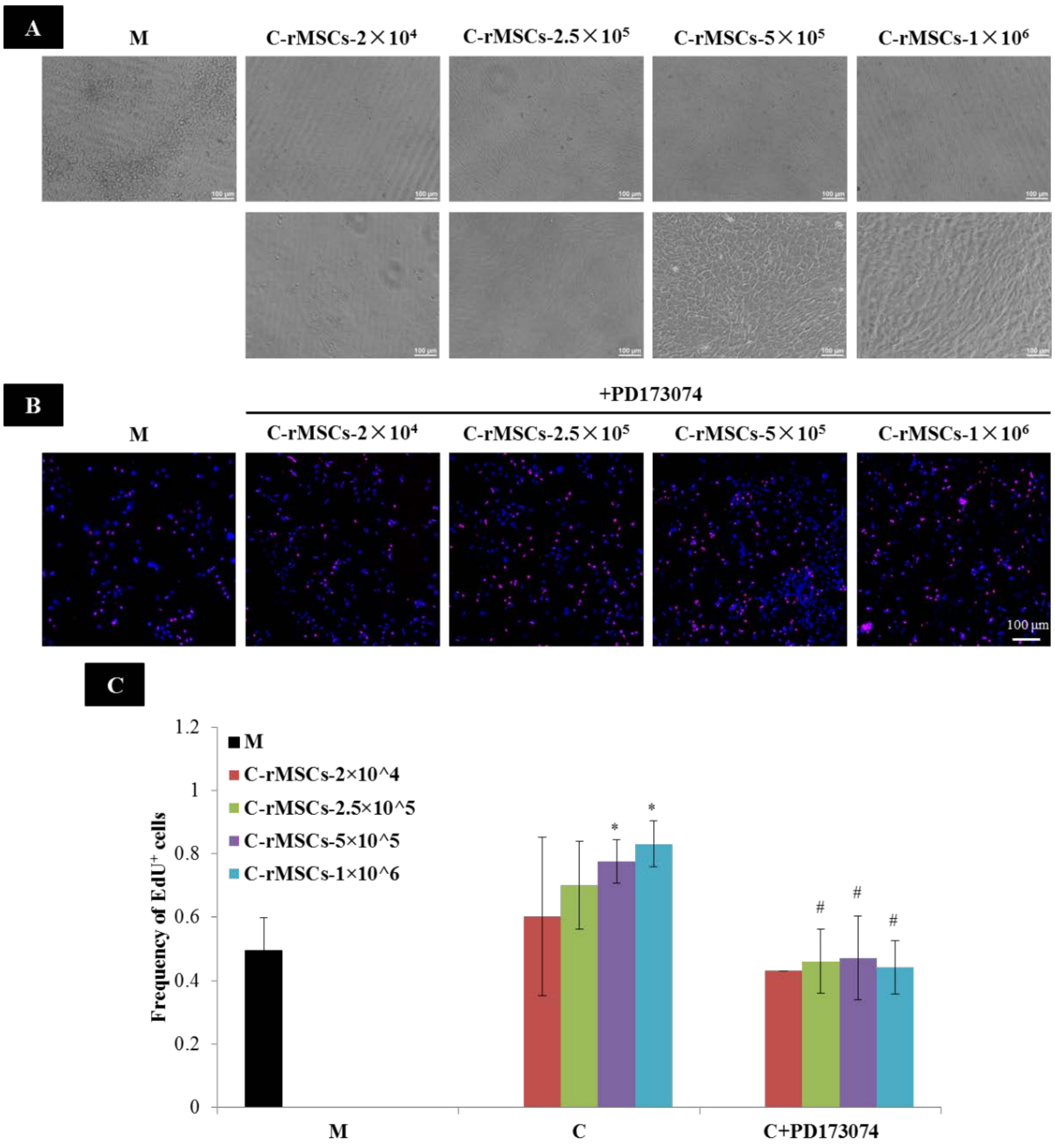

Figure S6. Coculture with PD173074 supplemented. (**A**) Cell morphology (day 6), (**B**) EdU assay (24 h), and (**C**) frequency of EdU^{+} cells (24 h). Morphology of rACs retained round at the presence of PD173074 and cell proliferation was inhibited in coculture with PD173074 supplemented. **M**: rACs monoculture in growth medium as control; **C-rMSCs-2×10^{4}**, **-2.5×10^{5}**, **-5×10^{5}** and **-1×10^{6}**: rACs cocultured with rMSCs at 2×10^{4}, 2.5×10^{5}, 5×10^{5} and 1×10^{6} cells/well, respectively; **+PD173074** or **C+PD173074**: PD173074 supplemented in coculture; **-PD173074** or **C**: no PD173074 supplemented in

coculture; *: $p<0.05$, compared with **M**; #: $p<0.05$, compared with **-PD173074**; n=3.

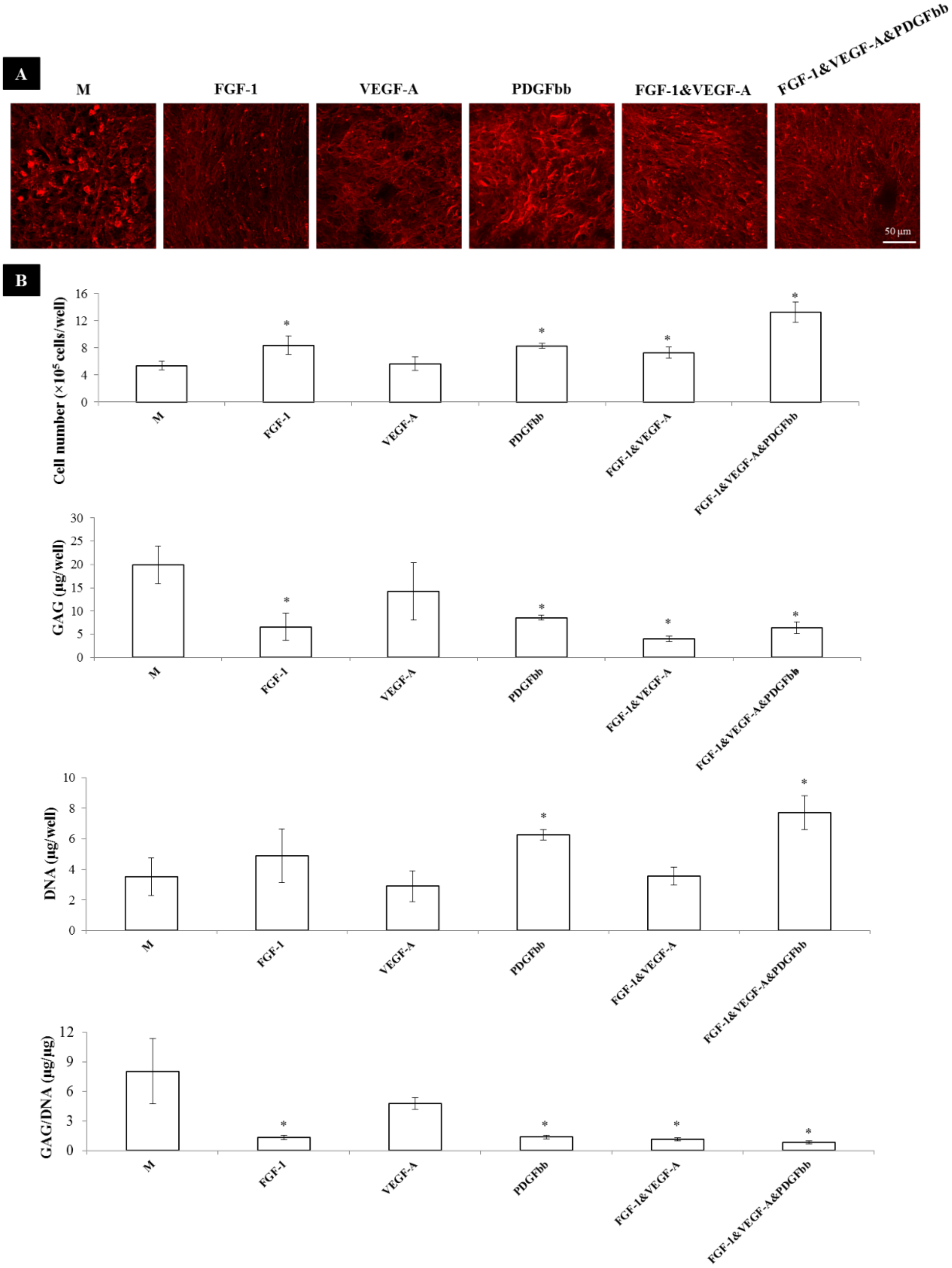

A
M
FGF-1
VEGF-A
PDGFbb
FGF-1&VEGF-A
FGF-1&VEGF-A&PDGFbb
50 µm
B
Cell number (×10^5 cells/well)
GAG (µg/well)
DNA (µg/well)
GAG/DNA (µg/µg)

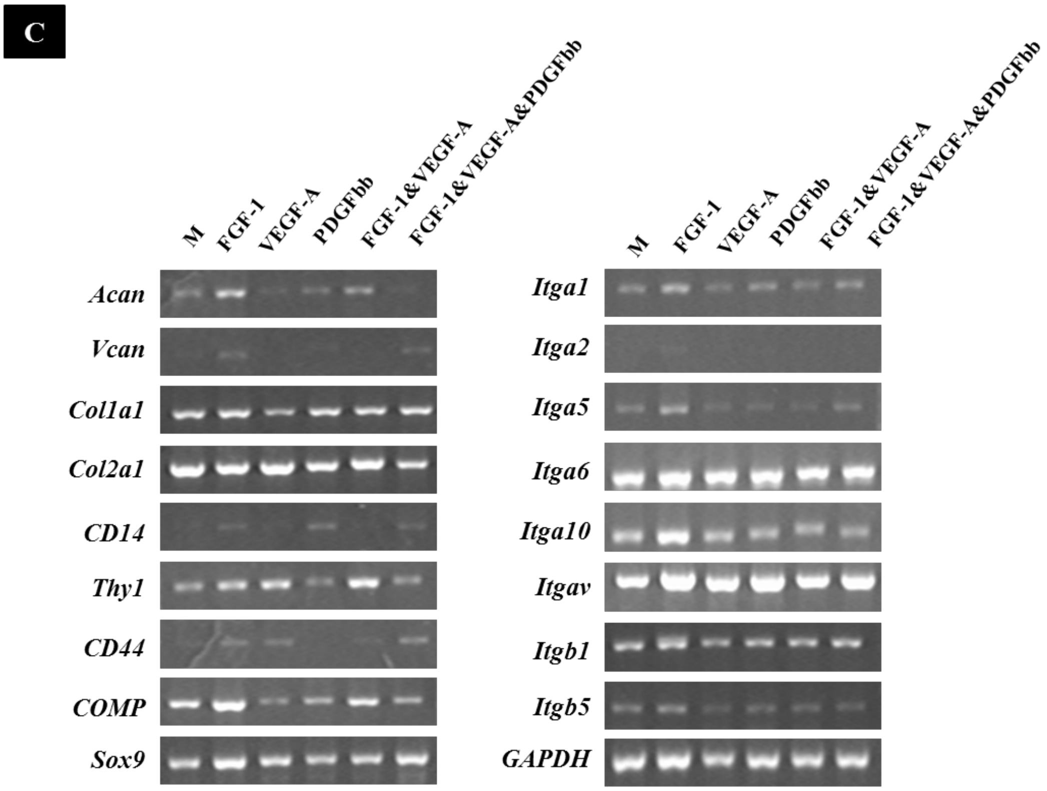

Figure S7. Grow factor supplementation at an increased dose in rACs monoculture. rACs were plated in 5×10^3 cells/cm^2 in chondrocyte growth medium and after 1 day, the medium was supplemented with either FGF-2 (10 ng/mL), VEGF-A (20 ng/mL), PDGFbb (20 ng/mL), FGF-2 (10 ng/mL) & VEGF-A (20 ng/ml) or FGF-2 (10 ng/mL) & VEGF-A (20 ng/ml) & PDGFbb (20 ng/mL). Culture lasted for 6 days. (**A**) F-actin staining (day 6), (**B**) cell number, DNA and GAG content (day 6), and (**C**) gene expression (day 4). A similar trend, but with more intensified effects was noticed, compared to that at the low dose (Figure 6). **M**: rACs monoculture without growth factors supplemented as control; *: p<0.05, compared with **M**; n=3

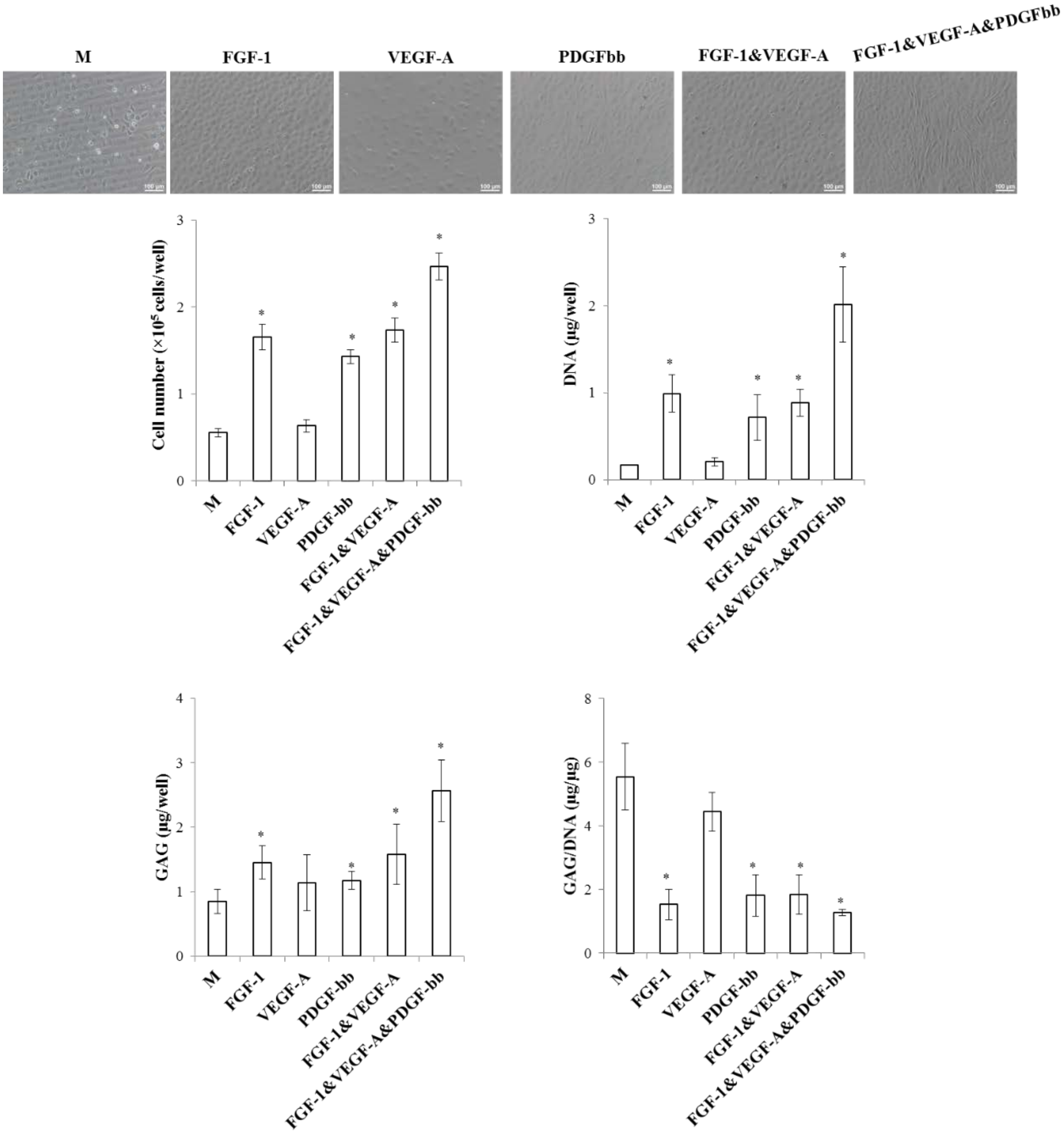

Figure S8. Monoculture of rACs in serum-free medium supplemented with grow factors. After 6 days of culture, cell morphology were observed under phase contrast microscopy, and cell number, DNA and GAG contents were quantified. Elongated cells could be observed with PDGFbb and FGF-1&VEGF-A&PDGFbb supplemented. Cell number was higher and GAG/DNA was lower with FGF-1, PDGFbb, FGF-1&VEGF-A and FGF-1&VEGF-A&PDGFbb supplemented compared to control. **M**: rACs

monoculture without growth factors supplemented as control; *: $p<0.05$, compared with **M**; n=3.
